# Inducing the Abscopal Effect in Liver Cancer Treatment: The Impact of Microwave Ablation Power Levels and PD-1 Antibody Therapy

**DOI:** 10.3390/ph16121672

**Published:** 2023-11-30

**Authors:** Changli Liao, Guiyuan Zhang, Ruotong Huang, Linyuan Zeng, Bin Chen, Haitao Dai, Keyu Tang, Run Lin, Yonghui Huang

**Affiliations:** 1 Department of Interventional Radiology, The First Affiliated Hospital, Sun Yat-sen University, No.58 Zhongshan 2nd Road, Guangzhou 510080, China; liaochli@mail2.sysu.edu.cn (C.L.); zhanggy36@mail2.sysu.edu.cn (G.Z.); zengly35@mail2.sysu.edu.cn (L.Z.); chenbin23@mail.sysu.edu.cn (B.C.); daiht3@mail3.sysu.edu.cn (H.D.); tangky6@mail.sysu.edu.cn (K.T.); 2 Department of Interventional Therapy, Sichuan Clinical Research Center for Cancer, Sichuan Cancer Hospital & Institute, Sichuan Cancer Center, Affiliated Cancer Hospital of University of Electronic Science and Technology of China, No. 55 South Renmin Road, Section 4, Chengdu 610041, China; 3 Department of Metabolism, Digestion, and Reproduction, Faculty of Medicine, Imperial College London, London SW7 2AZ, UK; r.huang23@imperial.ac.uk

**Keywords:** liver cancer, abscopal effect, microwave ablation, PD-1, immunotherapy

## Abstract

Microwave ablation (MWA) is an effective treatment for liver cancer (LC), but its impact on distant tumors remains to be fully elucidated. This study investigated the abscopal effects triggered by MWA treatment of LC, at different power levels and with or without combined immune checkpoint inhibition (ICI). We established a mouse model with bilateral subcutaneous LC and applied MWA of varied power levels to ablate the right-sided tumor, with or without immunotherapy. Left-sided tumor growth was monitored to assess the abscopal effect. Immune cell infiltration and distant tumor neovascularization were quantified via immunohistochemistry, revealing insights into the tumor microenvironment and neovascularization status. Th1- and Th2-type cytokine concentrations in peripheral blood were measured using ELISA to evaluate systemic immunological changes. It was found that MWA alone, especially at lower power, promoted distant tumor growth. On the contrary, combining high-power MWA with anti-programmed death (PD)-1 therapy promoted CD8^+^ T-cell infiltration, reduced regulatory T-cell infiltration, upregulated a Th1-type cytokine (TNF-α) in peripheral blood, and inhibited distant tumor growth. In summary, combining high-power MWA with ICI significantly enhances systemic antitumor immune responses and activates the abscopal effect, offering a facile and robust strategy for improving treatment outcomes.

## 1. Introduction

Liver cancer (LC) is a global health crisis, ranking as the sixth most common cancer by incidence and the third leading cause of cancer-related deaths worldwide [1]. Surgical radical treatment is recommended for only a minority of patients in the early disease stage [2]. In response to this daunting challenge, ablation has emerged as a cornerstone of clinical practice, offering an attractive option for localized LC treatment. Among ablation techniques, microwave ablation (MWA) has been consolidated as a first-line option for clinicians due to its safety and minimally invasive nature [3,4,5].

While MWA represents a promising approach in the domain of localized tumor control, particularly within the context of hepatocellular carcinoma (HCC), its impact on distant micrometastatic lesions, situated beyond the confines of the primary tumor milieu, remains enigmatic. Hepatocellular carcinoma (HCC), as a primary type of LC, is characterized by multifocal tumor development and micrometastases, factors that significantly complicate patient prognoses [6]. Facciorusso et al. demonstrated that even in patients with HCC in the early/intermediate stage, with the adoption of radiofrequency ablation (RFA) for curative purposes, the survival rate was only 52% at 5 years. The median post-recurrence survival (PRS) was 22 months. The median time to recurrence (TTR) from the first RFA performed was 38 months. There were 64 recurrence cases out of 103 treated patients, and only 34.3% were contiguous to or within the treated area. This means that the majority of recurrences should be recognized as “de novo” tumors rather than residuals of the initial ablated lesion. In other words, those undetected microscopic lesions have a profound impact on postoperative recurrence and overall prognosis [7]. Local therapy, as a monotherapy, depends mainly on the abscopal effect to produce the inhibition of non-treated lesions. This enigma stems from the intricate interplay between the local thermal ablative mechanisms of MWA and the intricacies of the ensuing immune responses. Upon administration, MWA instigates a sequence of coagulative necrosis, culminating in the demise of neoplastic cells, while concomitantly engendering a cascade of antigenic release at the ablation site [4,7,8]. The recruitment of antigens holds the key to the hypothesis that the activation of antitumor immune responses is potentially endowed with the capacity to exert a distal influence on micrometastatic lesions. It is a proposition that portends transformative implications for the oncological therapeutic landscape. However, there are counterargument suggestions regarding the inflammatory factors produced during thermal ablation, such as interleukin-6 (IL-6) and vascular endothelial growth factor (VEGF) [9,10]. Therefore, the abscopal effect induced by locoregional treatments, such as ablation or radiation therapy, becomes pivotal to the issue of whether the overall benefit can be achieved by choosing a localized treatment for HCC. Paradoxically, these immune effectors, though central to orchestrating immune responses, can inadvertently foster tumor progression. Thus, it becomes a dilemma that while MWA administration can set the stage for immune-mediated tumor quiescence, it also harbors the potential to inadvertently stoke the flames of neoplastic growth.

Meanwhile, recent investigations have spotlighted a pivotal variable regarding the power and temporal parameters characterizing MWA administration [9]. Their findings illustrate an intriguing revelation that the specific combination of the MWA power level and the duration of administration may serve as a decisive modulator in the generation of inflammatory mediators. Intriguingly, their observations suggest the potential of a resolution: high-power combined with abbreviated-duration MWA methodologies might offer a cogent strategy for mitigating this undesired phenomenon, offering promise in the complex landscape of considerations [9].

The abscopal effect, a phenomenon initially discovered in radiotherapy, holds the remarkable ability of localized treatments to induce regressions in distant metastases, a process intricately tied to immune responses [8,9]. This effect holds the potential to revolutionize the treatment of distant micrometastases. Prior studies have successfully unveiled the abscopal effect of thermal ablation in the treatment of malignancies, such as RFA and cryoablation [4,10,11]. However, the abscopal effect generated by MWA has remained elusive, primarily due to MWA’s limited capacity to induce immune responses [10]. On the other hand, the sublethal thermal effect from thermal ablation has been shown to be an important factor in promoting tumor proliferation and metastasis [12]. Thus, the existing literature presents conflicting evidence regarding the impact of ablation. While ablation can potentially induce immune-mediated tumor quiescence, it also carries the risk of unintentionally promoting neoplastic growth, particularly in studies involving MWA. Consequently, the effectiveness of MWA in multifocal tumors remains a dilemma.

In the landscape of cancer treatment, the advent of programmed death receptor-1 (PD-1) inhibitors has heralded a new era of promise in recent years [13,14]. These inhibitors function by enhancing the cytotoxic capabilities of T lymphocytes through the alleviation of immune cell inhibition within tumors [15]. The effectiveness of PD-1 blockade therapy is closely correlated with the level of antitumor T-cell immune responses within the tumor microenvironment [16,17]. Specifically, PD-1 blockade therapy boosts the activity of CD8^+^ T cells, which are essential for attacking cancer cells, and mitigates the suppressive effects of regulatory T cells (Tregs) [18]. This dual effect can contribute to a more favorable immune microenvironment for antitumor responses. However, in HCC, the outcomes have not been as promising. The Keynote-240 clinical trial showed some benefit in terms of overall survival, but the response rate was relatively low [19]. The CheckMate-459 trial demonstrated the benefit did not reach statistical significance compared to sorafenib [20]. Many experts attribute the unsatisfactory performance of ICI monotherapy to the unique and incoherent immune microenvironment of HCC [21]. Contrastingly, following the remarkable success of the combination of atezolizumab and bevacizumab in the IMBrave150 study, treatment strategy has been trending to a pattern of “ICI+” [22]. Clinical trials with high impact include LEAP002 (lenvatinib + pembrolizumab), the Himalaya study (durvalumab + tremelimumab), the EMERALD-1 study (TACE + durvalumab), and IMbrave 050 (atezolizumab plus bevacizumab after resection/ablation), and inspiring results have been achieved [23,24,25,26].

Therefore, combining PD-1 blockade therapy with MWA in the treatment of HCC is grounded in a carefully considered rationale that draws upon the complementary mechanisms of the two modalities. When administered alongside MWA, PD-1 inhibitors can augment the activity of immune cells that have been primed by the released tumor antigens [18]. This combination unleashes a more potent and coordinated antitumor immune response. The synergy between MWA and PD-1 blockade therapy has also been demonstrated to hold the potential to induce the elusive abscopal effect [27]. Although MWA alone has not traditionally been associated with inducing the abscopal effect due to its limited immune-inducing capacity, the addition of PD-1 inhibitors may tip the balance in favor of immune-mediated responses [10,27]. Meanwhile, recent investigations have spotlighted a pivotal variable regarding the power and temporal parameters characterizing MWA administration [28]. However, it is important to note that comprehensive studies specifically examining the combined impact of thermal ablation, particularly MWA, at different powers with anti-PD-1 therapy remain relatively scarce in the current literature.

Consequently, these revelations underscore the urgent need to untangle the intricate web of MWA’s influence on distant tumors. By investigating the intricate interplay between MWA and the immune system, particularly the potential impact on distant micrometastases, we aim to unravel the enigmatic relationship between localized tumor control and systemic antitumor immune responses. Through careful examination of MWA power levels and duration, we intend to determine optimal parameters that could minimize any unintended tumor-promoting effects while harnessing its antitumor potential. Moreover, we seek to explore the elusive abscopal effect in the context of MWA, which holds the promise of revolutionizing the treatment of distant micrometastases, often considered a significant challenge in liver cancer management. Additionally, by integrating PD-1 inhibitors into the treatment paradigm, we aim to enhance the immune-mediated response, potentially triggering the abscopal effect. The anticipated impact of this research lies in its potential to refine liver cancer treatment strategies, providing clinicians with valuable insights into optimizing MWA and PD-1 therapy combinations for improved patient outcomes, especially in cases involving multifocal tumors and micrometastases. Ultimately, this research has the potential to pave the way for innovative and more effective approaches for enhancing LC treatment outcomes and addressing this global health crisis.

## 2. Results

### 2.1. Enhanced Efficacy of High-Power MWA with Anti-PD-1 Treatment in Suppressing Distant Tumor Growth

**Figure 1:**
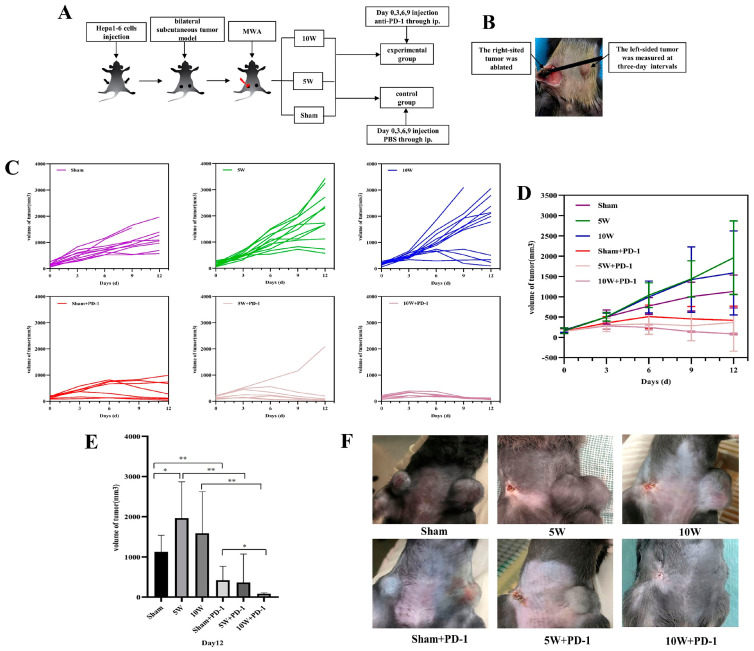
The schedule of the experiment and the growth curve of the left-sited tumor after MWA at different powers with or without anti-PD−1. (**A**) The schedule of the experiment. (**B**) The right-sited tumor was ablated, and the left-sited tumor was measured at three-day intervals. (**C**) The growth curve of the left-sited tumor of each mouse in each group from day 0 to 12; (**D**) the total growth curve of the left-sited tumor in each group from day 0 to 12; (**E**,**F**) the volume of the left-sited tumor on day 12. * indicates *p* < 0.05, ** indicates *p* < 0.01. MWA: microwave ablation; red lightning represents MWA; right-sided tumor with red represents the change after MWA; PBS: phosphate-buffered saline; ip: intraperitoneal injection.

In this study, we employed a murine model featuring bilateral subcutaneous tumors induced by hepa1-6 injection. The experimental protocol adhered to the scheme illustrated in Figure 1A,B. Figure 1C depicts the left tumor growth curve of each mouse in each group. The growth curve of the left tumor showed that there was no significant difference between the six groups on day 0 (Figure 1D). On day 12, the tumor volume of the 5W group was significantly larger than that of the Sham group (1961.55 ± 906.90 vs. 1128.87 ± 407.64, *p* = 0.035) (Figure 1E,F). Although the tumor volume of the 10W group was larger than that of the Sham group, the difference was not statistically significant (1586.58 ± 1036.20 vs. 1128.87 ± 407.64, *p* = 0.278). There was no significant difference between the 5W and 10W groups (1961.55 ± 906.90 vs. 1586.58 ± 1036.20, *p* = 0.399).

After combined treatment with anti-PD-1, it was found that the distant tumor growth was significantly inhibited in all combination groups (Figure 1D). On day 12 (Figure 1E,F), the distant tumor volume of the 10W + PD-1 group was significantly smaller than that of the Sham + PD-1 group (83.36 ± 22.88 vs. 419.44 ± 350.73, *p* = 0.037). There was no significant difference between the 10W + PD-1 group and the 5W + PD-1 group (83.36 ± 22.88 vs. 364.33 ± 707.48, *p* = 0.350). Moreover, the variations in the *p*-values pertaining to left tumor volume on day 12 post-ablation, as presented in Table S1, suggest that the introduction of PD-1 antibodies resulted in consistently diminished distant tumor volumes across all combination groups, in contrast to the control groups receiving PBS administration.

### 2.2. Enhanced CD8^+^ T-Cell Infiltration in Distant Tumors with High-Power MWA and Anti-PD-1 Therapy

**Figure 2:**
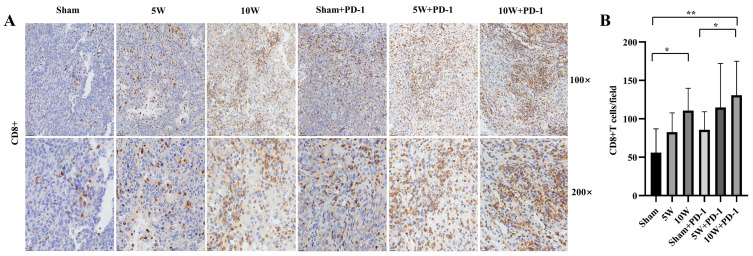
CD8^+^ T lymphocytes were detected by immunohistochemistry in the left tumor. Hematoxylin staining resulted in blue-colored nuclei, while positive staining showed a brown color. (**A**) The immunohistochemical images at 100× and 200× magnification. (**B**) A comparison of the number of CD8^+^ T lymphocytes in each high-magnification field in different groups. * indicates *p* < 0.05, ** indicates *p* < 0.01.

Our data acquired in the immunohistochemical analysis showed the number of infiltrating CD8^+^ T lymphocytes in the 10W group was significantly higher than that in the Sham group (110.75 ± 29.04 vs. 56.03 ± 30.89, *p* = 0.036), but there was no significant difference between the 5W group and the Sham group (82.56 ± 25.22 vs. 56.03 ± 30.89, *p* = 0.197). There was no significant difference between the 10W and 5W groups in the number of CD8^+^ T lymphocytes (110.75 ± 29.04 vs. 82.56 ± 25.22, *p* = 0.212) (Figure 2). When combined with anti-PD-1, the number of infiltrating CD8^+^ T lymphocytes in each combination group was further increased, and the number of infiltrating CD8^+^ T lymphocytes in the 10W + PD-1 group was significantly higher than that in the Sham group and the Sham + PD-1 group (130.74 ± 44.38 vs. 56.03 ± 30.89, *p* = 0.009; 130.74 ± 44.38 vs. 85.66 ± 23.53, *p* = 0.048). The difference in the number of infiltrating CD8^+^ T lymphocytes between the 10W + PD-1 and 10W groups was not statistically significant (130.74 ± 44.38 vs. 110.75 ± 29.04, *p* = 0.484). The number of infiltrating CD8^+^ T lymphocytes in the 5W + PD-1 group (114.97 ± 57.17) was higher than that in the Sham group, the 5W group, and the Sham + PD-1 group, but the difference was not statistically significant (*p* = 0.062, *p* = 0.304, *p* = 0.268). There was no significant difference in the number of CD8^+^ T lymphocytes between the 10W + PD-1 and 5W + PD-1 groups (*p* = 0.603) (Figure 2).

### 2.3. Combination Therapy Significantly Diminishes Treg Infiltration in Distant Tumors

**Figure 3:**
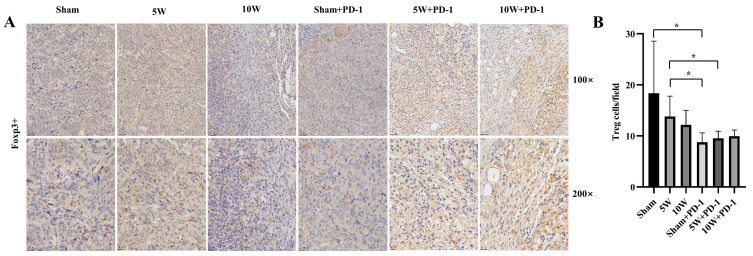
Regulatory T cells (Tregs) were detected by immunohistochemistry in the left tumor. Hematoxylin staining resulted in blue-colored nuclei, while positive staining showed a brown color. (**A**) The immunohistochemical images at 100× and 200× magnification. (**B**) A comparison of the number of Tregs in each high-magnification field in different groups. * indicates *p* < 0.05.

The extent of Treg infiltration within the Sham group (18.37 ± 10.20), the 5W group (13.80 ± 3.98), and the 10W group (12.15 ± 2.87) exhibited no statistically significant distinctions *(p* > 0.05), as depicted in Figure 3.

However, the number of Treg infiltrations was reduced when combined with anti-PD-1. The number of Treg infiltrations in the Sham + PD-1 group was significantly lower than that in the Sham group (8.77 ± 1.83 vs. 18.37 ± 10.20, *p* = 0.042) and the 5W group (8.77 ± 1.83 vs. 13.80 ± 3.98, *p* = 0.023). The number of Tregs in the 5W + PD-1 group was also significantly lower than that in the 5W group (9.54 ± 1.38 vs. 13.80 ± 3.98, *p* = 0.038). There was no significant difference between the 10W + PD-1 group and the Sham group (9.97 ± 1.39 vs. 18.37 ± 10.20, *p* = 0.068) and the 10W group (9.97 ± 1.39 vs. 12.15 ± 2.87, *p* = 0.146) (Figure 3).

### 2.4. Low-Power MWA Increases Tumor Microvessel Density in Distant Tumors

**Figure 4:**
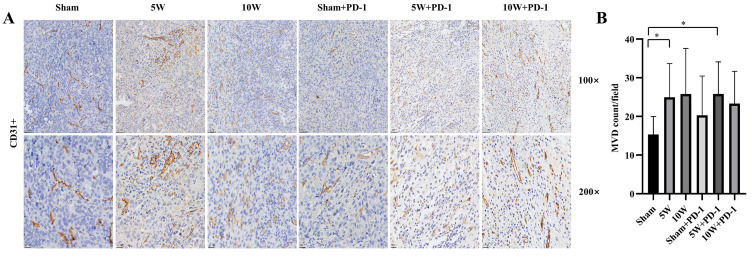
Microvascular density (MVD) was detected by immunohistochemistry in the left tumor. Endothelial cells stained in brown or clusters of endothelial cells were counted as single-countable microvessels. (**A**) The immunohistochemical images at 100× and 200× magnification. (**B**) A comparison of the number of microvessels in each high-magnification field in different groups. * indicates *p* < 0.05.

The number of microvessels in the 5W group was significantly higher than that in the Sham group (24.96 ± 8.71 vs. 15.36 ± 4.62, *p* = 0.048). There was no significant difference between the 10W group and the Sham group in the number of microvessels (25.80 ± 11.80 vs. 15.36 ± 4.62, *p* = 0.093) (Figure 4). Microvessel counts remain unaffected in response to combination with anti-PD-1 therapy. The number of microvessels in the 5W + PD-1 group was also significantly higher than that in the Sham group (25.83 ± 8.30 vs. 15.36 ± 4.62, *p* = 0.025), and there was no significant difference between the 5W + PD-1 group and the 5W group (25.83 ± 8.30 vs. 24.96 ± 8.71, *p* = 0.881). In a similar vein, the 10W + PD-1 group displayed comparable microvessel counts to both the Sham group (23.31 ± 8.37 vs. 15.36 ± 4.62, *p* = 0.064) and the 10W group (23.31 ± 8.37 vs. 25.80 ± 11.80, *p* = 0.722), with no statistically significant differences observed. Furthermore, no statistically significant differences in MVD were noted when comparing the Sham + PD-1 group with the other groups (*p* > 0.05) (Figure 4).

### 2.5. Enhanced TNF-α Levels in Peripheral Blood Achieved with Combined High-Power MWA and Anti-PD-1 Therapy

MWA alone has a limited impact on peripheral blood cytokine levels detected by ELISA. MWA monotherapy did not lead to significant alterations in the concentrations of Th1-type cytokines, TNF-α and IFN-γ, or Th2-type cytokines, IL-4 and IL-10, in peripheral blood.

**Figure 5:**
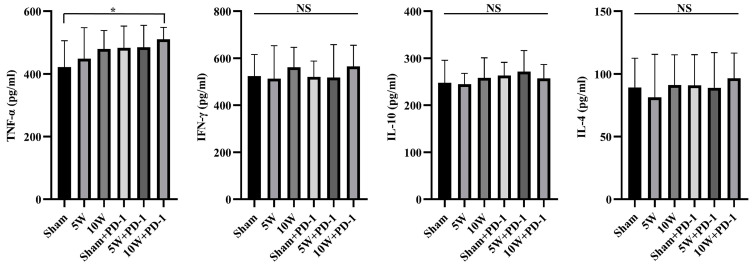
Cytokine TNF-α, IFN-γ, IL-10, and IL-4 concentrations in peripheral blood were detected by ELISA on day 12. * indicates *p* < 0.05, NS: not significant.

However, following the combination of MWA with anti-PD-1 therapy, it is noteworthy that only the 10W + PD-1 group demonstrated a statistically significant increase in the concentration of TNF-α in peripheral blood when compared to the Sham group (422.09 ± 83.93 vs. 510.63 ± 38.04, *p* = 0.033). In contrast, there were no significant differences observed among the 10W group, Sham + PD-1 group, 5W group, 5W + PD-1 group, and Sham group. There were no significant differences in the concentrations of Th1-type cytokine IFN-γ and Th2 cytokines IL-4 and IL-10 in peripheral blood among the three groups (Figure 5).

## 3. Discussion

The observation of low expression of PD-1/PD-L1 in LC has significant implications for the effectiveness of PD-1/PD-L1 inhibition as a monotherapy strategy [21]. PD-1/PD-L1 inhibition has demonstrated remarkable success in various cancer types, especially those with high expression of these immune checkpoint molecules, including non-small-cell lung cancer and melanoma [29,30]. However, in LC, the outcomes have not been as promising, and this discrepancy is evident in the results from clinical trials like the KEYNOTE and CheckMate trials. The Keynote-240 clinical trial investigated the use of pembrolizumab, a PD-1 inhibitor, in patients with advanced HCC who had previously been treated with sorafenib [19]. While the trial showed some benefit in terms of overall survival, the improvement was modest, and the response rate was relatively low. Similarly, the CheckMate-459 trial evaluated nivolumab, another PD-1 inhibitor, as a first-line treatment for advanced HCC [20]. While this trial demonstrated a trend toward improved survival compared to sorafenib, the benefit did not reach statistical significance. Together, these trials revealed that nearly 80% of patients with HCC did not respond effectively to anti-PD-1 monotherapy [19,20]. Recent preclinical studies have also confirmed that nearly 70% of HCC can be categorized as “non-inflamed class”, which means that these types of HCC have various degrees of immune escape and immune tolerance by different mechanisms [21,31]. Our experiment data appear to diverge from the findings of these phase III clinical studies; the Sham + PD-1 group exhibited more significant inhibition of distant tumor growth compared to the Sham group, which corresponds with the outcomes of an earlier study. Interestingly, the results from a phase 1/2 trial known as CheckMate-040 showed the safety and efficacy of nivolumab in patients with HCC [32]. To some extent, our study aligns with the findings of this trial. This implies that these variances might not always manifest as tangible clinical advantages, notwithstanding the statistical distinctions observed in treatment responses. From bench to bedside, numerous factors can influence the realization of clinical efficacy, including many cytokines. These cytokines include TNF-α, IFN-γ, IL-4, and IL-10. Even slight fluctuations in their concentrations and ratios can either enhance or impede the efficacy of anti-PD-1 treatments. In conjunction with our non-significant findings pertaining to CD8^+^ T cells, Treg infiltration, MVD, and the concentrations of TNF-α, IFN-γ, IL-4, and IL-10, these results underscore the challenges of using PD-1 inhibition as a monotherapy in LC, where low PD-1/PD-L1 expression may limit the ability of the immune system to mount an effective antitumor response. Consequently, it becomes imperative to investigate methodologies of greater clinical significance to attain the overarching objective of enhancing patient survival. Also, the immune microenvironment in HCC, characterized by low PD-1/PD-L1 expression, may considered as a restriction factor for the immune system to mount an effective antitumor response. Therefore, in order for PD-1 or PDL-1 inhibitors to be effective in boosting the T-cell immune response against HCC cells, it is necessary to combine them with other means to pursue the goal of amplifying the immunotherapeutic effect, for example, by evoking immunogenic cell death (ICD) to achieve the modification of the immune microenvironment. The modification of the tumor microenvironment in HCC is regarded as the trigger for the booster effect of PD-1 or PDL-1 inhibitors, and locoregional therapy has been proven to be an effective means of evoking ICD [33,34]. Therefore, MWA, despite being considered as having a relatively poor capacity for immune stimulation, can still remain a worthwhile option for research [35].

The clinical preference for MWA as a first-line treatment in LC stems from its well-established safety profile and minimally invasive nature [4]. MWA offers several advantages compared to other thermal ablation methods, including enhanced convection profiles, consistent intratumoral temperature control, faster ablation durations, and the capacity to simultaneously treat multiple lesions using multiple probes. MWA should be considered as the technique of choice when the tumor is ≥3 cm in diameter or is close to large vessels, independent of its size [36]. However, the clinical landscape of LC is characterized by its multifocal nature, with tumors often appearing at multiple sites within the liver [6]. Any locoregional therapy utilized in the clinic, including MWA, can only treat detectable foci and cannot target all multicentric lesions. Sequential progression of these multifocal lesions after ablation became the major source of relapse after the initial intervention. The PRS of distant site type were also proved to be significantly shorter than it of the treated site type [7].

Notably, in the case of nasopharyngeal carcinoma, despite the low expression of PD-1/PD-L1, local radiotherapy has been observed to induce the shrinkage or regression of distant metastases, a phenomenon known as the abscopal effect [37]. A study of surgical specimens of HCC revealed a significantly higher incidence of both microvascular invasion and micrometastases in tumors when the tumor size is >5 cm. This finding implied that most patients with HCC already have experienced microvascular invasion or micrometastases at the time of diagnosis [38]. Therefore, whether the abscopal effect can be triggered by locoregional therapy becomes pivotal to the therapeutic benefit. The abscopal effect occurs when local therapy not only shrinks the targeted tumor but also leads to the shrinkage of untreated tumors elsewhere in the body. Although the precise biological mechanisms responsible for the abscopal effect are still being investigated, the immune system is thought to play an important role [39]. Corresponding to the macro-physiological abscopal phenomenon is the micro-mechanistic ICD, and many locoregional treatments have been shown to induce the ICD effect, such as TACE and ablation [40]. However, in previously published studies, MWA was found to only induce such indirect effects and antitumor immune response in rare cases. A review of preclinical studies showed that the abscopal effect of RFA alone was seen in two of nine studies, while the abscopal effect of MWA alone was not seen in either of the two studies [41]. Huang et al. found that MWA alone failed to produce a significant abscopal effect, but the combination of MWA and immunotherapy resulted in the inhibition of distant tumor growth and reduced recurrence [27]. On the other hand, Yu reported that MWA not only suppressed tumor growth in the primary tumors but also stimulated an abscopal effect in CT26-bearing mice via the improvement of systemic and intratumoral antitumor immunity [42]. These studies implied that although MWA, like other ablative therapies, has an increase in interleukin-1 and heat shock proteins during cancer cell death via thermal injury, MWA is less inducible compared to radiofrequency ablation and cryoablation [35,43]. Moreover, Takaki et al. demonstrated that MWA possesses the capacity to inadvertently promote neoplastic growth [44]. Partially consistent with these statements, in this study, 5W MWA mice experienced significantly larger tumor growth than the control group, while there was no notable difference observed in the 10W group compared to the control. This seemingly contradictory information stems from the complex interplay between the localized thermal ablation mechanism of MWA and the ensuing complex immune response. Upon administration, MWA instigates a sequence of coagulative necrosis, culminating in the demise of neoplastic cells, while concomitantly engendering a cascade of cytokine release at the ablation site [4,33,45]. The release of various cytokines and its subsequent triggering of antigenic recruitment are key to the hypothesis that the activation of antitumor immune responses is potentially endowed with the capacity to exert a distal influence on micrometastatic lesions. However, there are arguments that cytokines produced during thermal ablation, such as interleukin-6 (IL-6) and vascular endothelial growth factor (VEGF), promote tumor proliferation and metastasis [28,46]. Thus, paradoxically, these immune effectors, though central to orchestrating immune responses, can inadvertently foster tumor progression. This inherent complexity poses a significant challenge, as MWA primarily serves as a locoregional treatment and cannot independently address the multifocal tumor development and micrometastases.

The abscopal effect has been noticed and studied as early as the 1950s, initially in the field of radiation therapy [47]. In recent years, with the clinical application of immunotherapy, the abscopal effect has regained emphasis. Since it is uncommon in part because cancer cells have ways to prevent the immune system from finding and killing tumor cells, ICI therapies exactly provide a means of overcoming the barriers to the immune response. Intriguingly, despite the multifocal nature of liver cancer that necessitates locoregional therapy to produce this effect, there was little evidence of ablation as a monotherapy inducing the abscopal effect in previous reports, especially not for MWA. As we enter the immunotherapy era, emphasizing the distinct challenges associated with LC treatment regarding how to harness the latent potential of locoregional therapies to induce abscopal effects becomes essential. Recognizing the multifaceted challenges posed by LC, a comprehensive and multidisciplinary approach that combines MWA with other therapies is imperative. In Table S2, the results of preclinical studies on locoregional therapy combined with ICI for HCC in recent years are reviewed and presented. Among them, three studies were on the combination of MWA and ICI. Although these studies differed in ablation power and degree of necrosis, they all showed that local therapy combined with ICI could significantly enhance the response of distal tumor inhibition. On the one hand, this demonstrates that ICI therapy is the cornerstone of the effective control of distant tumors, and on the other hand, it also implies that MWA is a possible means that can be considered to promote and enhance immune effects. In this study, we found that combining MWA with ICI strategies, PD-1/PD-L1 inhibitors, holds the promise to enhance the systemic antitumor immune responses required for distant tumor control. In contrast to the control groups, the introduction of anti-PD-1 resulted in consistently diminished distant tumor volumes across all combination groups. Furthermore, it is worth noting that the existing literature has proposed that the optimization of MWA parameters, particularly regarding timing and power, may potentially impede the growth of distant tumors [28]. These findings illustrate an intriguing revelation that the specific combination of the MWA power level and the duration of administration may serve as a decisive modulator in the generation of inflammatory mediators. Consequently, our study also delves into the investigation of the potential impact of varied MWA power levels and durations on the abscopal effect. We observed significantly reduced distant tumor volumes in the 5W + PD-1 and 10W + PD-1 groups compared to the Sham group, suggesting an augmented effect of anti-PD-1 therapy by MWA. However, while the distant tumor volume in the 5W + PD-1 group was smaller than that in the 5W group, it did not significantly differ from the Sham + PD-1 group. Notably, a significant disparity in tumor volume was evident between the 10W + PD-1 and Sham + PD-1 groups. These findings underscore the conditionality of enhancing the anti-PD-1 effect through MWA. These observations suggest the potential of a resolution: high-power plus abbreviated-duration MWA methodologies might offer a cogent strategy for mitigating this undesired phenomenon, offering promise in the complex landscape of considerations [28,48]. The choice of modality for locoregional therapy should be a pivotal detail valued as a trigger for the enhanced abscopal effect of PD-1 blocker therapy.

**Figure 6:**
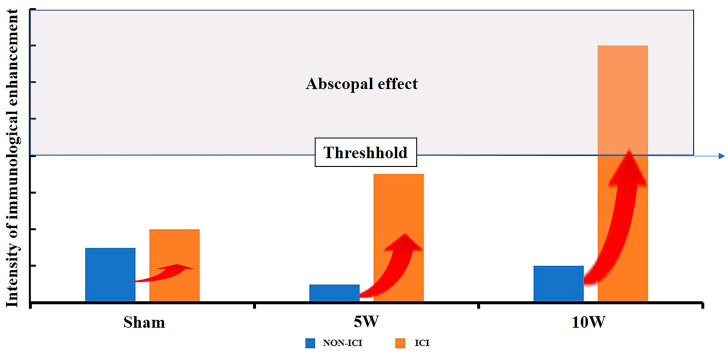
The threshold of the abscopal effect. When MWA was combined with ICI, Sham expressed relatively weak immune-enhancing intensity and 5W expressed moderate immune-enhancing intensity. Only 10W MWA showed obvious immune-enhancing intensity.

As to the underlying mechanism of the abscopal effect induced by MWA + anti-PD1, current studies have considered that ICD might be the explanation for the antitumor impacts. In addition, MWA has also been reported to induce ICD in osteosarcoma and to increase the proportion of CD8^+^ T cells. Furthermore, depletion of CD8^+^ T cells reversed the antitumor effects of MWA, indicating that CD8^+^ T cells play a key role in reducing osteosarcoma cells [49]. In addition, CTLA-4 and PD-1 are important checkpoint pathways that maintain T-cell activation, and blocking these two pathways contributes to T-cell reactivation and tumor rejection. When MWA is combined with anti-CTLA-4/PD-1 therapy, an increase in the CD8^+^ T-cell population may facilitate the clearance of unablated tumor cells, thereby protecting the host from tumor recurrence or neoplastic cells [50]. In our immunohistochemistry assays, the significant role of CD8^+^ T lymphocytes in antitumor immune responses was observed, with 10W MWA leading to a substantial increase in their infiltration, aligning with previous reports of thermal ablation increasing CD8^+^ T lymphocytes [4,10]. Additionally, 5W MWA showed a trend toward promoting CD8^+^ T lymphocyte infiltration; the extent was close to Sham + PD-1 and was not significant in comparison to Sham. Combining MWA with anti-PD-1 therapy further boosted CD8^+^ T lymphocyte numbers, especially in the 10W + PD-1 group, corroborating findings that PD-1/PD-L1 blockade promotes T lymphocyte infiltration [33]. Conversely, Tregs, known to limit antitumor immune responses, did not significantly decrease after treatment of MWA alone [51]. By combining anti-PD-1 therapy with MWA, we found a reduction in Treg numbers in distant tumors, suggesting potential synergistic effects. Maintaining the balance between Th1 and Th2 cytokines is crucial in tumor immunity, as Th1 cytokines are favorable for antitumor responses, while Th2 cytokines tend to hinder them [52,53,54]. In our study, high-power MWA combined with anti-PD-1 significantly increased the level of TNF-α, which is known for its direct tumor-killing effect, with no significant differences in the IFN-γ, IL-4, and IL-10 levels [55]. MWA including MWA of different power levels, or anti-PD-1 alone showed limited impact on TNF-α concentration; only 10W MWA in combination with a PD-1 inhibitor produced a relatively significant elevation. In brief, our results revealed that high-power MWA combined with anti-PD-1 significantly elevated the CD8^+^ T cell to Treg ratio in tumor tissue and increased the level of TNF-α in peripheral blood, suggesting that combined therapy with certain sophisticated matches holds the promise to enhance the systemic antitumor immune responses which are required for distant tumor control. Our study did not compare whether PD-1 expression was altered in distal tumors before and after MWA ablation. In a study of primary human colorectal tumors, Shi et al. reported that RFA treatment of liver metastases not only increased T-cell infiltration but also increased PD-L1 expression in primary human colorectal tumors [33]. Another preclinical study of MWA in combination with ICI by Guo et al. provides more direct evidence that MWA can lead to the overexpression of PD-1 and PD-L1 [56]. These studies, together with the increase in tumor volume after 5W MWA treatment alone in our study, strongly implicate that MWA ablation of one tumor among multiple liver cancers concurrently alters the tumor microenvironment of distant tumors by producing a cascade of cytokines and modulating the ratio of cytokines among them. All of these changes tend to be more favorable to ICI effectiveness. In our findings, what warrants further study is that high-power MWA with anti-PD-1 is highly capable of facilitating the abscopal effect. In a recent in vitro study on thermal ablation, researchers analyzed the changes in the concentrations of ICD-related cytokines produced by different HCC tumors at different temperatures, including ATP, HMGB1, and CXCL10, and the results indicated that extremely low temperatures (−80 °C) and higher temperatures (60 °C) are more conducive to facilitating the ICD effect [57]. Other real-world data from clinical liver cancer patients present changes in cytokines in the peripheral blood of patients under different energy levels of MWA ablation. The serum levels of IL-2 at 24 h post-MWA and IL-6 at 15 d post-MWA were positively correlated with energy output during the MWA procedure [58]. Those studies, in addition to confirming our findings that higher power is more effective in triggering the abscopal effect, also implied the hypothesis that there is a latent regular correlation between temperature, MWA power, and ICD-related cytokines. When these cytokines change to reach a certain threshold, they will trigger a synergistic effect with ICI treatment, thus allowing us to observe the abscopal effect (Figure 6). Our further studies will focus on the changes in ICD-related cytokines in tumor cells under different MWA powers to fully understand the various cytokine changes related to tumor progression and the immune microenvironment induced by different MWA powers. Based on the results of the current study, one of our important findings is that whether it is the abscopal effect on the macrophysical phenomenon or the ICD effect on the micro-mechanism, the power level of MWA has been identified as a dominant agent of the capacity to trigger an immune response to LC.

Apart from its impact on tumor-associated immunity, MWA also influences proliferation-related molecules. Elevated MVD is a well-established marker associated with tumor progression, metastasis, and patient prognosis [59]. Previous studies have indicated that thermal ablation can increase MVD both locally and in distant tumor sites [28,60]. In our study, we observed an increase in MVD in distant tumors following MWA, with a more significant increase in the low-power ablation group. However, the combination with anti-PD-1 did not mitigate the MVD increase induced by MWA, possibly due to the slower heating process of low-power MWA, which exposes marginal tumors to sublethal temperatures for longer durations [12]. This finding reaffirms our deduction that alterations in cytokines caused by MWA can be harnessed to both amplify immunotherapeutic efficacy and foster tumor proliferation.

The limitations of this study should be mentioned. Firstly, we did not monitor PD-1 expression levels in distant tumors before and after MWA ablation. Serum VEGF, a cytokine directly contributing to elevated MVD, was not examined in this study. Secondly, we only selected post-ablation serum to study the relevant cytokines, and we did not monitor the pattern of cytokine changes before and after ablation. There are also more ICD-related cytokines that have not been measured in this study. Due to the limited amount of fresh serum and tumor samples suitable for testing collected from the same pool of small experimental animals, it was difficult to complete numerous tests simultaneously. In the following study, we will further enrich the relevant data.

## 4. Materials and Methods

### 4.1. Study Design

Mice were subcutaneously injected with 1 × 10^6^ Hepa1-6 cells in the bilateral flank. Upon reaching a tumor size of 6–8 mm, the mice were subjected to random allocation into six groups according to the following design: Sham ablation group: Sham group; low-power group: 5W group; high-power group: 10W group; sham ablation combined with anti-PD-1 group: Sham + PD-1 group; low-power combined with anti-PD-1 group: 5W + PD-1 group; high-power combined with anti-PD-1 group: 10W + PD-1 group (Table 1). Each group consisted of a minimum of seven mice. Following the complete ablation of the right tumor, the size of the left tumor was measured at three-day intervals to assess the presence and extent of the abscopal effect. The left specimens were obtained for immunohistochemistry on the 12th day after ablation to analyze the variations in the immune microenvironment and neovascularization in the distant tumor. The peripheral blood was drawn on the 12th day for ELISA testing to determine the systemic immunological state. The experimental protocol was approved by the Animal Ethics Committee of Sun Yat-sen University (SYSU-IACUC-2021-000370).

### 4.2. Cell Line and Culture

Mouse hepatoma cell line Hepa1-6 was cultured in DMEM medium at 37 °C in a 95% air and 5% CO_2_ incubator (i150c, Thermo Fisher Scientific, Waltham, MA, USA) supplemented with 10% FBS and 1% penicillin/streptomycin [61].

### 4.3. Animal Model and Treatments

Male C57BL/6 mice, aged six to eight weeks, were utilized in this study. Hepa1-6 cells, at a concentration of 1 × 10^6^, were subcutaneously injected into symmetrical sites on both bilateral flank regions of the mice. Once the tumors reached a diameter of 6–8 mm, MWA (MTC-3C VISON MEDICAL) treatment was performed on the right-sided tumor. According to the previous study and preliminary experiment [62], complete ablation was performed using 5 watts of power for 3 min or 10 watts of power for 1.5 min. In sham ablation, the ablation needle was inserted into the tumor and directly pulled out.

PD-1 antibody (BE0273, BioXCell) treatment was given by intraperitoneal injection at a dose of 2 mg/kg at 30 min, day 3, day 6, and day 9 after MWA in the experimental group, whereas mice treated with MWA alone were injected with 2 mg/kg of PBS by i.p. injection in the control group [45].

### 4.4. Tumor Evaluation

The abscopal effect was assessed by measuring the longest diameter (L) and the shortest diameter (S) of the left tumor with a Vernier caliper every three days after MWA [27]. The tumor volume was calculated according to the following formula:

V = (L × S^2^)/2

### 4.5. Histopathologic Examination

The left tumor was fixed in a 10% formalin solution, followed by embedding in paraffin. Slices with a thickness of 4 μm were obtained from the paraffin-embedded tumor specimens. Subsequently, the slices were subjected to staining with hematoxylin and eosin. Anti-CD8 (diluted 1:1000, GB114196, Servicebio, Wuhan, China), anti-CD31 (diluted 1:600, GB11063-2, Servicebio, Wuhan, China), and anti-FOXP3 (diluted 1:500, GB11093, Servicebio, Wuhan, China) antibodies were detected using immunohistochemistry.

Hematoxylin staining resulted in blue-colored nuclei, while positive staining showed a brown color. The quantification of CD8^+^ and FOXP3^+^ cells was performed by observing the number of cells in five randomly selected areas under magnifications of 100× and 200×. The results were averaged to obtain a representative value [45].

MVD was examined under 100× and 200× magnification in five randomly selected sites. Endothelial cells stained in brown or clusters of endothelial cells were counted as single countable microvessels. Vessels with thick muscle walls or a lumen diameter exceeding the equivalent of 8 red blood cells (about 50 mm) were excluded from the analysis [63].

### 4.6. Enzyme-Linked Immunosorbent Assay

The blood was drawn through the ocular on the 12th day following treatment and left to stand at room temperature for coagulation. Serum was collected by centrifugation at 1000 RPM for 10 min and kept at −80° [45]. Th1-type TNF-α and IFN-γ and Th2-type IL-10 and IL-4 concentrations in serum were determined using an ELISA kit (Meimian Biotechnology, Yancheng, Jiangsu, China).

### 4.7. Statistical Analysis

SPSS Software (version 25, IBM, New York, NY, USA) and GraphPad Prism software (version 9, Boston, FL, USA) were used for statistical analysis and drawing. Continuous data are expressed as mean ± SD. One-way analysis of variance (ANOVA) was used for comparisons of groups. *p* < 0.05 was considered to indicate a significant difference.

## 5. Conclusions

In summary, our experimental results indicate that both 5W and 10W MWA can activate tumor immune-related communities, thereby enhancing the efficacy of ICI therapy. However, only the combination of 10W MWA with anti-PD-1 exhibited a significant abscopal effect. Our study revealed that the local treatment of one lesion can lead to dramatic changes in the microenvironment of the other distant tumor, which include some factors promoting tumor progression, and at the same time, certain factors can also be applied to enhance the overall therapeutic effect via the combination of ICI. Because the abscopal effect can only be achieved when the immune-enhancing effect reaches a certain level to counteract factors induced by MWA that promote tumor proliferation, in order to avoid the harms and obtain the benefit of the best therapeutic response, reasonable ablation power selection should be considered as critical for triggering the abscopal effect.

## Figures and Tables

**Table 1:** The groups and the treatments involved in the study.

	Sham MWA	Low-Power MWA	High-Power MWA
PBS	Sham	5W	10W
Anti-PD-1	Sham + PD-1	5W + PD-1	10W + PD-1

PBS: phosphate-buffered saline.

## Data Availability

The datasets used and analyzed in the current study are available from the corresponding author upon reasonable request.

## Supplementary Materials

The following supporting information can be downloaded at: https://www.mdpi.com/article/10.3390/ph16121672/s1.
