# Supplementary material for: Inducing the Abscopal Effect in Liver Cancer Treatment: The Impact of Microwave Ablation Power Levels and PD-1 Antibody Therapy
Source: Pharmaceuticals (Basel). 2023 Nov 30;16(12):1672. doi: 10.3390/ph16121672 (PMC10747918; doi:10.3390/ph16121672)
Supplement: Supplementary file 1 [file pharmaceuticals-16-01672-s001.zip › pharmaceuticals-2697406-supplementary.pdf]

Supplementary Materials:

Table S1. The difference in the P value of the left tumor volume between the groups on day 12.

	Sham	5W	10W	Sham+PD-1	5W+PD-1	10W+PD-1
Sham	1	**0.035**	0.278	**0.005**	**0.031**	**<0.001**
5W	-	1	0.399	**0.001**	**0.002**	**<0.001**
10W	-	-	1	**0.015**	**0.020**	**0.002**
Sham+PD-1	-	-	-	1	0.867	**0.037**
5W+PD-1	-	-	-	-	1	0.350
10W+PD-1	-	-	-	-	-	1

Statistically significant results are shown in bold, with "-" indicating coincidence with the corresponding group.

Table S2. Recent pre-clinical trial about the combination of ablation and immunomodulation

Author	Species	Complete ablation	Ablation modality	Power	Immunomodulator	Results
Zhong et al. (doi:10.1080/02656736.2020.1836406)	Mouse	Yes	MWA	5 w for 3 min	DCs injection	Enhance CTLs activities, decrease Treg number, reduceIL-10 production, induce INF-μ, inhibit the growth of distant tumor.
Huang et al.(doi:10.1080/02656736.2022.2032406)	Mouse	Yes	MWA	5 w for 3 min	Anti-PD-1 antibody	Enhance survival, inhibit growth of distant tumor, induced tumor-specific immune response, increase Th1 response, resist tumor rechallenge.
Duan et al.(10.1080	Mouse	Yes	MWA	5 w for 3 min	Anti-PD-1 antibody/a	Increase survival, resist tumor rechallenge,

/15384101.2020.1853942)					nti-CTLA4 body	enhance CLTs activities,increase Th1 response.
Sun et al. (doi:10.1016/j.biopha.2023.115351)	Mouse	iRFA (70 % necrosis and 30 % residual tumors)	RFA	70℃ for 2 min	OK-432/antiPD-1 antibody	Enhance CTLs activites, decrease Treg in residual tumor, increase survival, inhibit growth of residual tumor, resist tumor rechallenge.
Tan et al(doi:10.1016/j.apsb.2022.08.006)	Mouse	Yes	CRA/ MWA	2 freezing cycles /10 w for 30-60s	Anti-PD-1 antibody	CRA showed superior curative effect than MWA local tumor combining with anti-PD-L1 antibody by strengthening CTL/NK cell immune response.
DCs: dendritic cells; CTLs: cytotoxic T lymphocytes; Th1: type-1 helper T cells; RF: radiofrequency; CpG-oligodeoxynucleotides: Treg: regulatory T cells; CTLA-4: cytotoxic T lymphocyte associated protein 4; PD-1: programmed cell death 1; iRFA: incomplete RFA; CRA: cryoablation.						
